# Flexible Label-Free Platinum and Bio-PET-Based Immunosensor for the Detection of SARS-CoV-2

**DOI:** 10.3390/bios13020190

**Published:** 2023-01-26

**Authors:** Rodrigo Vieira Blasques, Paulo Roberto de Oliveira, Cristiane Kalinke, Laís Canniatti Brazaca, Robert D. Crapnell, Juliano Alves Bonacin, Craig E. Banks, Bruno Campos Janegitz

**Affiliations:** 1 Laboratory of Sensors, Nanomedicine and Nanostructured Materials, Federal University of São Carlos, Araras 13600-970, Brazil; 2 Department of Physics, Chemistry, and Mathematics, Federal University of São Carlos, Sorocaba 18052-780, Brazil; 3 Faculty of Science and Engineering, Manchester Metropolitan University, Chester Street, Manchester M1 5GD, UK; 4 Institute of Chemistry, University of Campinas, Campinas 13083-970, Brazil; 5 Department of Chemistry and Chemical Biology, Harvard University, Cambridge, MA 02138, USA

**Keywords:** flexible electrode, Bio-PET, platinum-based electrode, point-of-care, 3D printing technology, SARS-CoV-2 diagnostics

## Abstract

The demand for new devices that enable the detection of severe acute respiratory syndrome-coronavirus-2 (SARS-CoV-2) at a relatively low cost and that are fast and feasible to be used as point-of-care is required overtime on a large scale. In this sense, the use of sustainable materials, for example, the bio-based poly (ethylene terephthalate) (Bio-PET) can be an alternative to current standard diagnostics. In this work, we present a flexible disposable printed electrode based on a platinum thin film on Bio-PET as a substrate for the development of a sensor and immunosensor for the monitoring of COVID-19 biomarkers, by the detection of L-cysteine and the SARS-CoV-2 spike protein, respectively. The electrode was applied in conjunction with 3D printing technology to generate a portable and easy-to-analyze device with a low sample volume. For the L-cysteine determination, chronoamperometry was used, which achieved two linear dynamic ranges (LDR) of 3.98−39.0 μmol L^−1^ and 39.0−145 μmol L^−1^, and a limit of detection (LOD) of 0.70 μmol L^−1^. The detection of the SARS-CoV-2 spike protein was achieved by both square wave voltammetry (SWV) and electrochemical impedance spectroscopy (EIS) by a label-free immunosensor, using potassium ferro-ferricyanide solution as the electrochemical probe. An LDR of 0.70−7.0 and 1.0−30 pmol L^−1^, with an LOD of 0.70 and 1.0 pmol L^−1^ were obtained by SWV and EIS, respectively. As a proof of concept, the immunosensor was successfully applied for the detection of the SARS-CoV-2 spike protein in enriched synthetic saliva samples, which demonstrates the potential of using the proposed sensor as an alternative platform for the diagnosis of COVID-19 in the future.

## 1. Introduction

The SARS-CoV-2 pandemic outbreak was decreed by the World Health Organization (WHO) in March 2020 [1], significantly affecting especially underdeveloped countries, due to several factors, including the costs and/or availability of diagnostic tests for managing the health crisis, with early diagnosis being one of the most important factors in the identification, control, and treatment of infectious diseases. In overcrowded health systems, COVID-19 progression monitoring is also especially important, allowing healthcare providers to identify critical patients [2]. It is known that some preexisting health conditions, including cardiovascular diseases (CVs), are related to a worse prognosis of COVID-19 [3,4]. Studies have shown that the levels of some aminothiols, such as cysteine (Cys) and homocysteine (Hcy), in the blood are related to CVs, making them interesting molecules to monitor [5,6]. Furthermore, Hcy was recently cited as a potential relevant biomarker for COVID-19 progression [7] and its total blood level (t-Hcy) seems to be associated with the total blood level of Cys (t-Cys) in both control patients and patients with coronary heart disease [6]. Lastly, some studies show a decrease in Cys serum levels in patients infected with SARS-CoV-2 when compared with control patients, in low, medium, and high interleukin 6 (IL-6) levels [8]. Therefore, t-Cys blood levels might be an interesting potential complementary tool for aiding in COVID-19 diagnosis and/or progression monitoring, with L-Cys being the most abundant form of cysteine in our body. [9,10].

At present, COVID-19 diagnoses are mainly performed by reverse transcription-polymerase chain reaction (RT-qPCR) [11], enzyme-linked immunosorbent assay (ELISA) [12], and computed tomography imaging [13], among others. However, such analyses are commonly complex, expensive, and/or require highly trained technicians to handle the equipment. In this context, the search for diagnostic tests that can act as an alternative and that are rapid and low cost with the possibility of miniaturization has been increasing in academic society [14,15,16,17].

The use of electrochemical immunosensors can act as an alternative because these can be fast, low cost, portable, have high sensitivity and selectivity, as well as ease of application, including in clinical procedures in the non-invasive analysis [18,19]. Regarding the confection of sensors and immunosensors, the use of adequate conductive material and substrates are extremally important, taking into account their electrical, mechanical and thermal properties, as well as flexibility, biocompatibility, sustainability, and low cost [20,21].

The kind of conductive material used is important to the success of the analytical application from the electron transfer point-of-view. One example of a widely used conductive material is platinum (Pt) which shows high potential in the development of biosensors due to its high conductivity, catalytic properties, chemical inertia, and biocompatibility [22,23]. These characteristics are interesting for the efficient immobilization of biorecognition elements and for guaranteeing the bioactivity of the biomolecules. It is important to notice that Pt has similar properties to gold, presenting a strong interaction with thiol groups, such as the one present in cysteamine [24]. Mishra et al. [25] published a paper reporting a Pt-based electrochemical impedance immunosensor for human cardiac myoglobin. Functionalized Pt showed bioaffinity, which improved the bioelectrode properties in terms of stability and sensitivity. Another example of the use of Pt was reported by Li et al. [26] who detected alpha-fetoprotein. The immunosensor showed high sensitivity, presenting a linear range of 0.0001 to 100 ng mL^−1^ with an LOD of 0.028 pg mL^−1^.

Furthermore, the substrate and the technique used in the preparation of the electrodes allow the versatility of the devices obtained, which can be integrated into 3D platforms and used in point-of-care devices (POCDs). Among the substrates, bio-based polyethylene terephthalate (Bio-PET) is an attractive option because it is renewable, affordable, environmentally friendly, and has excellent gas and moisture barrier properties [27,28]. Andreotti et al. [29] developed a work for the determination of hydroquinone, epinephrine, and serotonin using PET bottles as the substrate for the preparation of the electrochemical sensor. The use of PET [30] provided excellent properties such as chemical inertness, dimensional stability, and good barrier properties against moisture and gases.

The selection of the sensor manufacturing process is also critical in device production taking into account reproducibility, scalability, reliability, and compatibility with substrate properties [31]. Many processes can be used for material deposition, such as pyrolysis spray, chemical vapor deposition, inkjet printing, sputtering, photolithography, and others [32,33,34]. In this sense, the photolithography technique is usually adopted to produce electrochemical (bio)sensors [35] because this strategy allows the attainment of thinner films, resulting in a more sensitive signal with lower LOD, and decreasing the film resistance. An interesting work using this deposition technique was described by Oliveira et al. [30] who used Pt-based electrodes by photolithography and an environmentally friendly substrate (Bio-PET) for the development of an immunosensor for the detection of Parkinson’s disease.

Electrochemical immunosensors can be applied in the monitoring of several (bio)clinical markers in a non-invasive way, such as saliva, sweat, and tears [36,37]. In addition, they allow for a cost-effective measurement compared with traditional clinical analysis techniques, such as ELISA and RT-PCR, for example. Their application commonly does not require special training or long periods for the obtention of results, allowing the examiner to perform the analysis easily and accurately. Allied with immunosensing, 3D printing appears as a great alternative tool for the development of new prototypes that allow the reduction of analytical steps, volume of samples, and cost [38,39,40]. In this work, a 3D-printed device was applied considering its properties as well as for the control of the electroactive sensor area.

The dual detection of biomarkers and their measurement using different detection systems paves the way for a new generation of sensors for highly sensitive, cost-effective, and rapid diagnostics. Biomarkers and their concentrations reflect biological processes and indicate the presence or severity of the corresponding diseases. However, due to the heterogeneous nature of most diseases, the types and concentrations of most discriminative biomarkers vary at different stages between individuals [41]. Therefore, the detection of a single biomarker alone can be insufficient for an accurate clinical diagnosis or to track the progression of diseases, such as COVID-19, for example.

Hence, in the present work, we propose an electrochemical sensor and immunosensor, using a printed electrode based on Bio-PET and Pt made by the photolithography technique for the detection of L-cysteine and the SARS-CoV-2 spike protein. Furthermore, to demonstrate the potential of using the proposed platform as POCDs, we prepared a portable 3D-printed device for analysis.

## 2. Materials and Methods

### 2.1. Reagents and Solutions

All reagents used in this work were of analytical grade. Ultrapure water (Milli Q, ^®^ Burlington, MA, USA), with resistivity >18 MΩ cm, was used to prepare all the aqueous solutions. We obtained potassium hexacyanoferrate (III) (K_3_[Fe(CN)_6_]) (>99.0%) from (Dinamica^®^, Goiania, Brazil), L-cysteine (>98.0%) from (Alfa Aesar, Ward Hill, MA, USA), methionine (>98.0%) from (Fisher BioReagents, USA), citric acid (Dinamica^®^, Brazil), urea (>99.0%) from (Vetec^®^, Duque de Caxias, Brazil), and tyrosine (>98.0%), caffeine (>99.0%), fructose (>99.0%), glucose (>98.0%), cysteamine (CYS) (>95.0%), bovine serum albumin (BSA) (>96.0%), and glutaraldehyde (GLA) from Sigma-Aldrich^®^, St. Louis, MO, USA. The recombinant SARS-CoV-2 spike protein and the SARS-CoV-2 spike antibody (Ab) were obtained from Sino Biological (Beijing, China). For the detection of L-cysteine, 0.10 mol L^−1^ PBS (pH 6.0) was used as dilution and supporting electrolyte solutions, which were prepared using sodium phosphate dibasic (Dinamica^®^, Brazil), potassium chloride, and sodium phosphate monobasic (Sigma-Aldrich^®^, USA). Phosphate-buffered saline solution (PBS 1x, pH 7.4) was prepared using potassium chloride (>99.0%), sodium chloride (>99.0%) from (Vetec^®^, Brazil), sodium phosphate dibasic (>98.0%), and potassium phosphate monobasic (>98.0%), from Cinetica, Brazil. Synthetic saliva was prepared with sodium chloride, sodium phosphate dibasic, sodium bicarbonate (>98.0%) from Labsynth, Brazil, potassium chloride, and urea.

### 2.2. Structural Characterization

The sensor surface evaluation was performed by scanning electron microscopy (SEM) using a Thermo Fisher Scientific Prisma E microscope with ColorSEM technology and integrated with an energy-dispersive X-ray spectrometer (EDS) for the chemical mapping of the electrode surface. The water contact angle experiments, before and after the modification of the immunosensor surface, were performed aiming to evaluate its hydrophobic or hydrophilic characteristics. The lab-made goniometer used for image acquisition was developed by the research group and has been described in the literature [42]. For image acquisition, the 3D-printed equipment was placed to couple a smartphone to photograph the drop onto the analyzed surface. The treatment of the images was done using the software CorelDRAW Graphics Suite 2019 (Corel Corporation, Canada).

### 2.3. Apparatus and Electrochemical Measurements

All electrochemical measurements were performed in an AutoLab PGSTAT204 potentiostat/galvanostat with an impedance module FRA32M managed by NOVA 2.1.4 software (Metrohm, Utrecht, Netherlands). Cyclic voltammetry (CV) measurements were performed from 0.0 to +1.2 V, at a scan rate of 50 mV s^−1^, and chronoamperometry measurements at an applied potential of +0.55 V were used for the determination of L-cysteine. On the other hand, electrochemical impedance spectroscopy (EIS) was recorded from 100 kHz to 0.10 Hz, with an amplitude of 10 mV, and square wave voltammetry (SWV) measurements were performed with an amplitude of 80 mV, a frequency of 80 Hz, and a step potential of 10 mV. EIS and SWV were applied for the detection of the SARS-CoV-2 spike protein. For these analyses, 0.10 mol L^−1^ KCl (pH 7.0) was used as a supporting electrolyte, and an equimolar mixture of 5.0 mmol L^−1^ [Fe(CN)_6_]^3−/4−^ was applied as a redox probe. The EIS measurements were calibrated by normalized impedance change (NIC%) [43,44] concerning the control. The NIC% value was calculated using Equation (1), where, in this case, *Zcontrol* is the magnitude of charge transfer resistance (Rct) for a control sample without the antigen, and *Zsample* is the magnitude of Rct for each added point of concentration of the antigen.

(1) $$NIC\%=\frac{Z_{sample}-Z_{control}}{Z_{control}}\times 100\;\;\;\;\;\;$$

#### D Device Fabrication for Measuring

**Figure 1:**
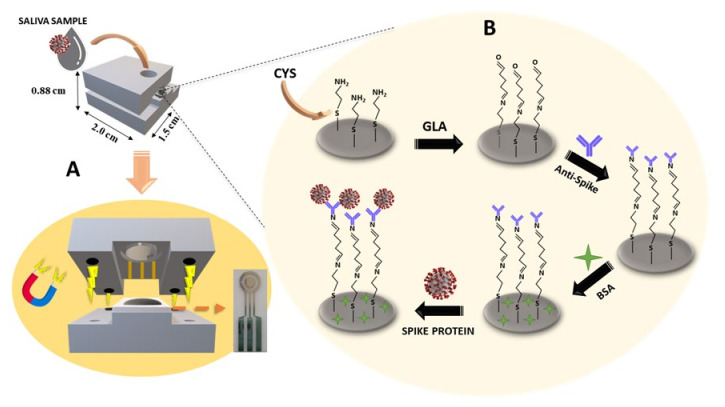
Representative schematic of the 3D-printed clinical analysis device in (**A**) and the steps involved in the manufacturing of the immunosensor in (**B**).

A 3D-printed clinical analysis device was designed in the software Blender version 2.91.2 and manufactured using a Sethi3D S3 3D printer employing polylactic acid (PLA) filament, as illustrated in Figure 1 (step A). The 3D printing was performed at extrusion and table-heated temperatures of 200 °C and 65 °C, respectively, with full infill. Additionally, the STL files for 3D printing are available on the journal website. The device was composed of two parts (base and cover) measuring 2.0 × 1.5 × 0.9 cm^3^ that allowed the coupling of the 3D-printed electrodes, containing a sample addition zone with electrode area and solution volume control of 10 µL (Figure S1 in Supplementary Materials). Four magnets were attached to each part of the support for the electrode fixation, which guarantees greater practicality in its use.

### 2.4. Immunosensor Fabrication

Flexible Pt electrodes (geometric area of working electrode 0.03 ± 0.1 cm^2^) were fabricated by conventional photolithography using Bio-PET sheets according to Oliveira et al. [30]. Figure S2 shows images of the flexible Pt electrodes on Bio-PET (Pt/Bio-PET). For the immunosensor, initially, 8.0 µL of an aqueous solution of cysteamine (CYS, 10.0 mmol L^−1^) was deposited on the surface of the working electrode at room temperature for 12 h. The electrodes were then washed with deionized water to remove excess non-immobilized CYS. Then, 8.0 μL of a solution of glutaraldehyde (GLA, 5.0 mmol L^−1^), prepared in 0.2 mol L^−1^ phosphate buffer (PB) pH 8.0, was deposited on the CYS-modified surface for 1 h, followed by a washing step with PB. An aliquot of 8.0 µL of 1.0 µmol L^−1^ anti-SARS-CoV-2 spike protein antibody (PBS 1×, pH 7.4) was added on the electrode surface for 2 h, followed by a washing step with PBS 1×. The electrodes were then washed with PBS 1× to remove excess material and incubated with 8.0 μL of BSA solution (1% w/v) in PBS 1× (pH 7.4) for 30 min to block the remaining binding sites [45], followed by washing with PBS 1×. After that, the immunosensor was washed with PBS 1× before being used in spike protein detection experiments. Figure 1 (step B) demonstrates a representative scheme of the steps involved in the assembling of the immunosensor. The immunosensor assays were performed by EIS and SWV measurements before and after allowing 8.0 μL of the spike protein solution (0.70 to 30 pmol L^−1^) to interact with the modified working electrode surface for 1 h. All procedures were performed at room temperature (25 °C).

### 2.5. Sampling Procedure for the Detection of SARS-CoV-2

Synthetic saliva samples were used to test the performance and practical application of the device. The synthetic saliva was prepared according to the procedure reported by Romonti et al. [46] and spiked with 7 concentration levels of the SARS-CoV-2 spike protein (0.7, 1.0, 3.0, 5.0, 7.0, 10, and 30 pmol L^−1^), which were within the linear working range of the immunosensor.

## 3. Results

### 3.1. Morphological and Electrochemical Characterization of the Pt/Bio-PET

**Figure 2:**
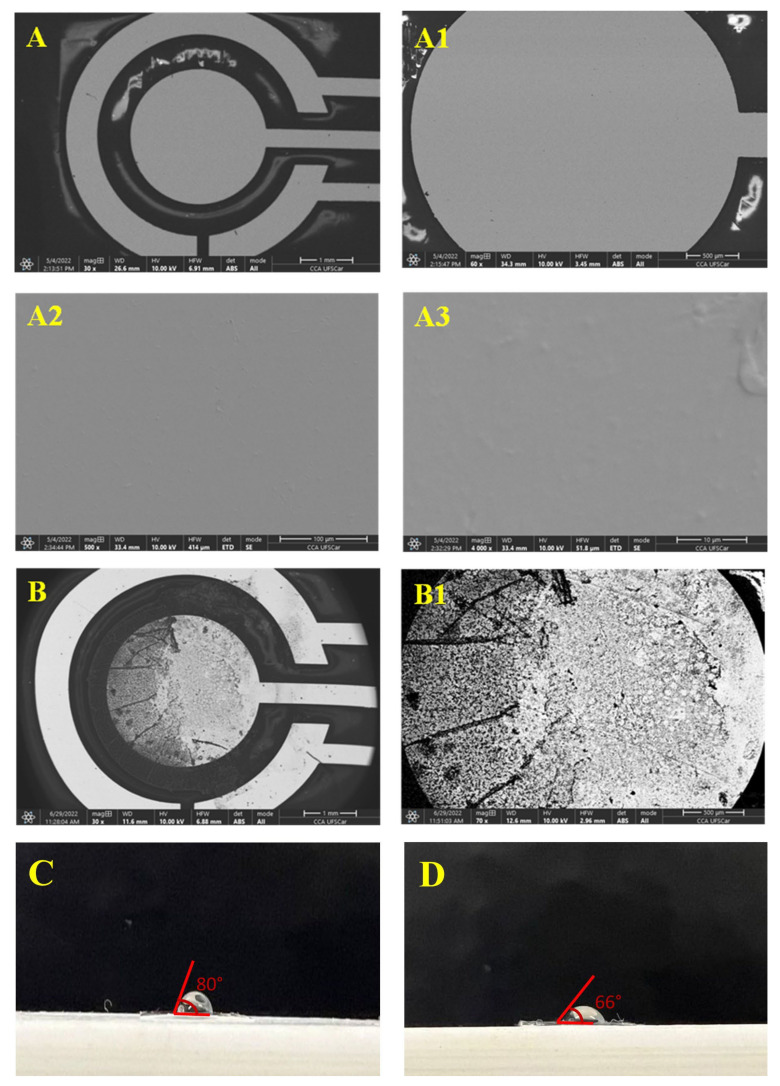
(**A**) SEM images of the platinum working electrode at different magnifications: 30× (**A**), 60× (**A1**), 500× (A2) and 4000× (**A3**), respectively. (**B**) SEM images of the modified platinum working electrode at different magnifications: 30× (**B**) and 60× (**B1**), respectively. Water contact angle images obtained for the electrodes (**C**) before and (**D**) after the modification.

The surficial characteristics of the Pt/Bio-PET were evaluated by SEM-EDS in two different strategies (using the 180° and 90° aluminium sample holders) to perform the analyses. For possible differences between the Pt/Bio-PET and the immunosensor (SARS-CoV-2-Ab/Pt/Bio-PET) surfaces, before and after the modification, the electrodes were placed on the 180° aluminium holder. The SEM images are shown in Figure 2A,B, and the EDS spectra can be found in Figure S3.

It is possible to observe the excellent homogeneity of the Pt film onto the Bio-PET substrate, even at different magnifications Figure 2A1–A3. The high homogeneity can ensure a better reproducibility of the electrodes in the antibody immobilization step, as well as guaranteeing a better charge transfer process in electrochemical measurements. Analyzing the SEM images of the electrode after modification with the antigen-antibody complex Figure 2B,B1, it is possible to observe the formation of a film onto the Bio-PET working electrode surface, suggesting the success of the modification step. The EDS chemical mapping showed the semi-quantitative estimation of the elements on the electrode, such as carbon, platinum, and chromium, which can be confirmed by the EDS spectra, before sensor surface modification (Figure S3), demonstrating the main elements of the electrode material manufactured by photolithography and sputtering techniques.

The presence of a small percentage of chromium is due to the thin layer (10 nm) which is added for subsequent adhesion of the platinum film, and the presence of aluminum is due to the used sample holder.

For the Pt film thickness evaluation, the Pt/Bio-PET was cut in the WE region to expose the conductive part, and the device was placed on the 90° aluminum sample holder. Figure S4 shows the thickness of the Pt film with a mean value for the working electrode of 212.2 ± 0.1 µm. This feature is interesting because thin films generally exhibit different properties than bulk materials since the surface-to-volume ratio is much higher for thin films. This allows thin films to have the high flexibility and high conductivity required for the development of electrochemical devices [47,48]. This also demonstrates that photolithography and sputtering techniques are attractive for the large-scale production of electrodes on Bio-PET substrates.

The contact angle was measured for the working electrode surface before (Figure 2A) and after modification (Figure 2B) to provide information on electrode surface modification. For this, 6.0 µL of deionized water was used to evaluate the hydrophobic or hydrophilic characteristics of the surface of the working electrode. The surface can be classified as super-hydrophilic (θ < 10°), hydrophilic (θ < 90°), hydrophobic (90° < θ < 150°), or superhydrophobic (θ > 150°) [42]. Therefore, the electrode surfaces before and after the modifications showed a hydrophilic character, presenting a contact angle of 80 ± 1° and 66 ± 1°, respectively. The decrease in the contact angle can be explained by the presence of cysteamine, which shows a more hydrophilic behavior in the water measurements [49], proving its presence on the electrode surface, which is interesting for the success of the immobilization of biomolecules in later stages.

Electrochemical characterizations were performed by cyclic voltammetry (CV) to evaluate the electrochemical behavior of the Pt electrode using 0.10 mol L^−1^ KCl and an equimolar mixture of 5.0 mmol L^−1^ [Fe(CN)_6_]^3–/4–^ as a redox probe. Figure S5A shows the CV obtained at a scan rate of 50 mV s^−1^. The *Ipa/Ipc* ratio was 1.03, indicating the reversibility of the redox process on the electrode surface. In addition, a high current magnitude can be observed for the Pt electrode, a characteristic arising from the metal electrodes [30,50]. The electroactive area of the Pt electrode was calculated by CV at different scan rates, ranging from 10 to 250 mV s^−1^ (Figure S4B). The electroactive area was calculated based on the Randles–Ševčík equation [51]. The inserted curve of Figure S5B, related to the correlation between the peak current versus sweep square root (ν^1/2^), shows a linear behavior for both surfaces with a linear response of R^2^ = 0.997, following the equation: Ipa (μA) = 1.79 × 10^−7^ + 2.39 × 10^−4^ ν^1/2^ (V s^−1^)^1/2^, which indicates that for the Pt electrode, the mass transport process is controlled by diffusion. The electroactive area of 0.31 ± 0.1 cm^2^ from the geometric area of 0.03 ± 0.1 cm^2^ was calculated. The increase in the value of the electroactive area to the geometric area may be related to the Pt film by sputtering deposition.

### 3.2. Electrochemical Detection of L-Cysteine

**Figure 3:**
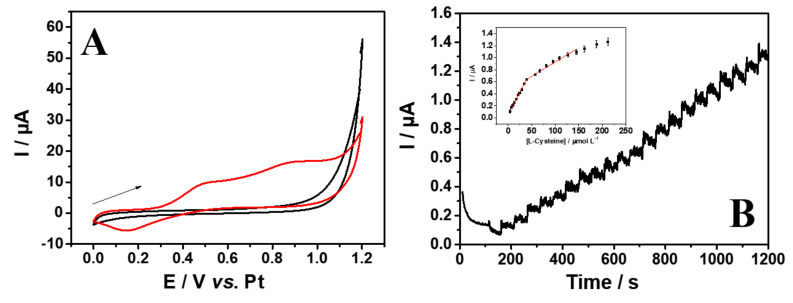
(**A**) Cyclic voltammograms in the presence (▬) and absence (▬) of 500 μmol L^−1^ L-Cys; scan rate of 50 mV s^−1^. (**B**) The analytical curve obtained by chronoamperometry for L-Cys (3.98−39.0 and 39.0−145 µmol L^−1^) by using the platinum electrode. Inset: Plot of peak current versus L-Cys. Applied potential of 0.55 V. Supporting electrolyte: 0.1 mol L^−1^ PBS pH 6.0.

L-cysteine has been suggested as a potential biomarker associated with the progression of SARS-CoV-2 disease [31]; thus, its detection is a potentially important tool for assessing the outcomes of COVID-19. Initially, the detection of L-Cys was evaluated by CV in 0.1 mol L^−1^ PBS (pH 6.0) in the presence of 500 μmol L^−1^ of L-Cys at 50 mV s^−1^ (Figure 3A).

The measurement in the presence of L-Cys has shown an irreversible behavior with two oxidation peaks, at 0.50 and 0.90 V (vs. Pt), and a reduction peak at 0.15 V (vs. Pt), which demonstrates the electroactivity of this species using the proposed electrode. Figure S6 demonstrates the L-Cys oxidation mechanism that occurs on the surface of the Pt electrode and is reported in the literature [52]. It is possible to observe that L-Cys is adsorbed on the electrode surface and oxidized by PtOH to sulfenic acid. The desorbed intermediate is oxidized by a disproportionation step to sulfinic acid. After that, the intermediate is oxidized to adsorbed sulfone and then oxidized to sulfonic acid species. The adsorbed sulfone can also be generated by the chemical oxidation of the adsorbed thiol by PtO.

For obtaining a better analytical response, voltammetric studies were undertaken to evaluate the influence of the pH of the solution on the electrochemical response. CVs in the presence of 500 μmol L^−1^ L-Cys were obtained in pH ranges from 5.0 to 9.0 (Figure S7). A decrease of the anodic peak currents (Ipa) for L-Cys oxidation (+0.55 V vs. Pt) can be observed with the pH increasing. This may be related to the ionization of Cys depending on the pH in aqueous solutions, as Cys contains several functional groups as –COOH, –SH, and –NH_2_ with pKa values of 1.92, 8.37, and 10.7, respectively [53]. Furthermore, the change in anodic and cathodic peaks with the increase in the pH is a consequence of the deprotonation involved in the oxidation process, which is facilitated at lower pH values. Another point to mention is that the isoelectric point of CySH is around 5.02; therefore, in strong acidic media, the L-Cys molecule is positively charged (CySH_2_^+^ (H_3_A^+^)), and increasing pH changes it to neutral or negative charges [53]. Thus, the PBS pH 6.0 supporting electrolyte was used for further studies because it is within the physiological pH range (6.0 to 8.0) and has a higher current and Ipa.

The influence of scan rate on the electrooxidation of L-Cys was investigated using the CV technique. Figure S8A shows the CV obtained for 1000 μmol L^−1^ L-Cys at scan rates ranging between 10 and 300 mV s^−1^. It is possible to observe the increase of the anodic peak current (Ipa) with the increasing scan rate. Ipa increases linearly proportional to the scan rate square root (ν^1/2^), with the regression equation Ipa (µA) = −1.19 × 10^–6^ + 9.43 × 10^–7^ ν^1/2^ (mV s^−1^)^1/2^ and R^2^ = 0.990, indicating that the oxidation process of L-Cys is diffusion-controlled [48], as expected for electrocatalytic systems [54,55].

Therefore, chronoamperometry under stirring conditions was used for the Pt electrode evaluation with successive injections of L-Cys at an applied potential of +0.55 V, which was sufficient to promote the oxidation of the molecule (Figure 3B). The selection of the lowest potential (first oxidation peak) resulted in a decrease in the oxidation of interfering species. Under successive additions in a 100 s time interval, a calibration curve was obtained for increasing concentrations of L-Cys. It is possible to observe two linear ranges for the detection of L-Cys, at concentrations from 3.98 to 39.0 µmol L^−1^ and from 39.0 to 145 µmol L^−1^ (Figure 3C). Linear regressions show I (μA) = 6.20 × 10^−8^ + 8.85 × 10^−9^ C_L-Cys_ (µmol L^−1^) with an R^2^ of 0.996 for the first linear range, and I (μA) = 4.54 × 10^−7^ + 1.04 × 10^−8^ C_L-Cys_ (µmol L^−1^) with an R^2^ of 0.990 for the second linear range. The limits of detection (LOD) and quantification (LOQ) were calculated based on the IUPAC definition (LOD = 3σ/s and LOQ = 10σ/s), where σ is the standard deviation of baseline noise and s is the analytical sensitivity of the calibration curve [56]. The values of LOD and LOQ obtained were 0.70 and 2.36 µmol L^−1^ (first linear range) and 2.19 and 7.31 µmol L^−1^ (second linear range).

The selectivity of the Pt/Bio-PET for the L-cysteine determination (50.0 µmol L^−1^) was evaluated in the presence of possible concomitant species found in biological fluids or pharmaceutical products (500 µmol L^−1^), such as methionine (MET), tyrosine (Tyr), caffeine (CAFF), citric acid (CA), urea, glucose (GLU), and fructose (FRUC). In the case of medical applications, the average concentration of methionine in the bloodstream is in the range of 5.0 to 10 µmol L^−1^ [57], the average concentration of fructose in the urine of healthy individuals is 23.0 µmol L^−1^ [58], and, after ingestion of food, it does not exceed 0.6 mmol L^−1^ in the bloodstream [59]. For tyrosine, its plasma concentration is normally in the range of 30 to 120 µmol L^−1^ [60], and for glucose, at normal levels, it can be found in human serum in the range of 4.0 to 6.0 mmol L^−1^ [61]. The other analytes may be present in pharmaceutical product formulations and their concentration may vary according to the product. Thus, the concentration of 1:10 (L-Cys: concomitant species) was studied to evaluate the maximum levels of each interference. It is possible to observe that after the addition of the interferents, the current response of L-Cys slightly decreased by 4.65% (Figure S9). This shows that the selectivity of the Pt/Bio-PET sensor for the detection of L-Cys has not been affected in the presence of the evaluated interfering molecules.

The proposed Pt/Bio-PET sensor was compared with other already reported electrochemical devices for the detection of L-Cys [57,62,63,64,65,66,67,68,69,70,71,72,73,74], as presented in Table S1. For example, Singh et al. [64] developed a sensor modified with reduced graphene oxide-cyclodextrin-platinum nanocomposites. The authors obtained an LOD of 0.12 µmol L^−1^ with a linear range of 0.5 to 170 µmol L^−1^. Although the LOD reported by the authors is slightly smaller than the one reported here, the preparation and confection of the material require many steps, which makes the sensor manufacturing process more time-consuming and more expensive. In another work, Atacan et al. [66] also proposed a graphene-based sensor for the detection of L-Cys. The reduced graphene oxide (rGO) sensor decorated with copper ferrite and gold nanoparticles displayed an LOD of 0.383 µmol L^−1^ with a linear range of 50.0 to 400 µmol L^−1^. The manufacturing steps of the sensor proposed by the authors also required several material preparation steps, and the use of macro electrodes, such as a glassy carbon electrode (GCE), as a substrate can restrict the sensor for point-of-care analysis. After that, Abbas et al. [57] developed a graphite paste sensor modified with iron nanoparticles and phthalocyanine. The sensor showed a linear range from 50.0 to 1000 µmol L^−1^ and an LOD of 0.27 µmol L^−1^. Although phthalocyanine with iron nanoparticles improves the catalytic effect for L-Cys detection, the use of graphite paste electrodes prepared by adding paraffin oil does not always guarantee the uniformity of the material, which can suffer sedimentation and thus vary the thickness of the membrane formed [75]. In addition, the proposed Pt/Bio-PET sensor presents as an advantage the ability to promote the oxidation and determination of L-cysteine without the use of catalysts or redox mediators, such as iron nanoparticles or Prussian blue.

### 3.3. Evaluation of the Electrochemical Performance of SARS-CoV-2-AB/Pt/Bio-PET as a Label-Free Immunosensor

**Figure 4:**
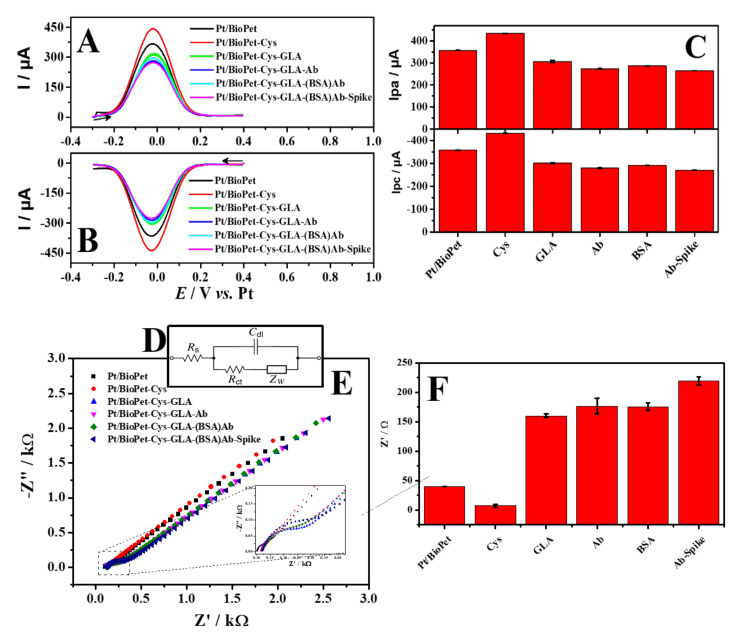
SWV voltammograms at (**A**) direct and (**B**) reverse. (**C**) Graph of peak current after each modification step, obtained from (**A**,**B**). (**D**) The equivalent circuit used for simulation of the experimental data, in the presence of redox couples: R_ct_, electron-transfer resistance; Rs, the resistance of the electrolyte solution; C_dc_, is the double-layer capacitance and; W, Warburg impedance. (**E**) Nyquist diagram (**F**) Graph of peak current after each modification step, obtained from (**D**). Platinum-printed electrode (Pt/BioPET), Pt/BioPET-cysteamine (Pt/BioPET-CYS), Pt/BioPET-CYS-glutaraldehyde (Pt/BioPET-CYS-GLA), Pt/BioPET-CYS-GLA-antibody (Pt/BioPET-CYS-GLA-Ab), Pt/BioPET-CYS-GLA-(BSA) antibody (Pt/BioPET-CYS-GLA-(BSA)Ab) and Pt/BioPET-CYS-GLA-(BSA) Ab-SARS-CoV-2 Spike Protein (Pt/BioPET-CYS-GLA-(BSA)Ab-Spike).

To evaluate the efficiency of the immunosensor assembly, SWV (forward and reverse scan) and EIS measurements were performed after each immobilization step, as well as for the immunosensor in the presence of the SARS-CoV-2 spike protein, using 5.0 mmol L^−1^ [Fe(CN)_6_]^3−/4−^ as an electrochemical probe (Figure 4). Initially, the effect of the presence of cysteamine (CYS) alone onto the Pt/Bio-PET surface was evaluated. When compared with the bare Pt/Bio-PET electrode, an increase in the peak intensity of the redox pair was observed for SWV, from ΔIpa = 72.89 μA and ΔIpc = 74.28 μA. On the other hand, for EIS, a decrease in the charge transfer resistance (Rct) was observed, from ΔRct = –32.76 Ω. This results from the electrostatic attraction between [Fe(CN)_6_]^3−/4−^ and the protonated amine groups of the cysteamine, leading to the increase in the current response (SWV) and the decrease in the Rct (EIS). The behavior has already been reported in the literature for the development of immunosensors [74,75,76,77].

Following this, GLA was used as a ligand to covalently bind the antibody on the electrode surface [68]. Carbonyl ends of the glutaraldehyde bind to the free primary amino groups of the cysteamine. At the other end of glutaraldehyde, the aldehyde group is free to bind with the amine group present in the antibody molecules. After the glutaraldehyde immobilization, it was possible to observe a significant decrease of the redox current intensity (SWV) and an increase of the R_ct_ value (EIS). This behaviour occurs because there is a partial blocking of the electrode surface after the species incorporation [78], which allowed the monitoring of each step in the construction of the immunosensor. This same behaviour was reported in the work of Mauruto et al. [30]. Next, SARS-CoV-2 antibodies (SARS-CoV-2-Ab) were immobilized on the surface of the Pt/BioPET-CYS-GLA, leading to a decrease in the redox current and the increase of R_ct_ since the SARS-CoV-2-Ab is a non-electroactive molecule for the electrochemical probe.

BSA was used to block active carboxylic acid groups and stabilize biomolecules already immobilized on the electrode surface (Pt/BioPET-CYS-GLA-(BSA)Ab). This reduces non-specific binding and avoids false-positive results in the analytical signal, leading to increased robustness of the sensor [79]. Immobilization of BSA showed no significant effect in the voltammetric (SWV) and impedimetric (EIS) responses.

Finally, the incubation of the antigen (SARS-CoV-2 spike protein)) (0.1 μmol L^−1^) led to a further decrease of the redox current of 264 µA for Ipa and from −270 µA for Ipc and an increase of R_ct_ of 219 Ω (Pt/BioPET-CYS-GLA-(BSA)Ab-Spike) due to the blocking of the electron transfer between the electrode surface and the redox probe, caused by the formation of the antigen-antibody complex [80]. Therefore, the decrease in the value and final redox current (SWV) and increase in Rct (EIS) proved that the formation of the immunocomplex was successfully performed, attesting to the functioning of the immunosensor. For the EIS technique, a simulation of the adjustment of the equivalent circuit was performed for the first and last step of immunosensor modification (Figure S10). From the results observed in the assembly of the immunosensor, it can be concluded that the studies by SWV and EIS show an excellent correlation between the results obtained after each modification step, indicating that both techniques can be used for further studies.

### 3.4. Electrochemical Determination of SARS-CoV-2 Spike Protein

**Figure 5:**
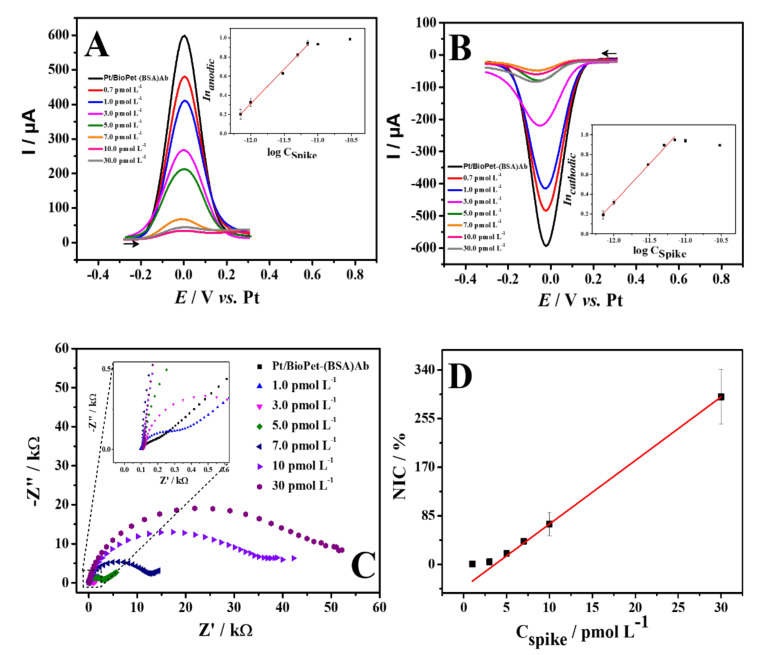
Square wave voltammograms direct (**A**) and reverse (**B**) obtained for different concentrations of spike protein of 0.7−7.0 pmol L^−1^. Inset: Plot of peak current versus spike protein concentration of 0.7−7.0 pmol L^−1^. SWV experimental conditions: square wave amplitude: 80 mV; frequency: 80 Hz; step potential: 10 mV. (**C**) Nyquist plots obtained for different concentrations of spike protein of 1.0−30 pmol L^−1^. (**D**) Plot of spike protein concentrations versus NIC%. Redox probe: 5.0 mmol L^−1^ [Fe(CN)_6_]^3−/4−^ in 0.10 mol L^−1^ KCl (pH7.0).

Aiming to verify the analytical performance of the proposed immunosensor, concentrations of spike protein were evaluated through SWV and EIS, and analyzed in terms of Ipa, Ipc, and NIC%. The influence of operating parameters (i.e., pulse amplitude, frequency, and step potential) in the electrochemical response was performed by using SWV. The obtained results are presented in Figure S11, and the values of the optimized parameters were square wave amplitude of 80 mV, step potential of 10 mV, and frequency of 80 Hz. Parameters were selected based on the highest current response value and peak definition. Under optimized parameters, SWV and EIS calibration curves were performed for increasing concentrations of the SARS-CoV-2 spike protein (Figure 5).

For SWV, a linear behavior was obtained over a concentration range from 0.7 to 7.0 pmol L^−1^, following the equations: In (anodic) = 9.02 + 0.725 logC_spike_ (pmol L^−1^), with R^2^ = 0.994, and In (cathodic) = 9.67 + 0.779 logC_spike_ (pmol L^−1^), with R^2^ = 0.993 (Figure 5A,B). This behavior in terms of the concentration in logarithmical form is similar to other works using voltammetric immunosensors [81,82] that describe an adsorptive effect for the antigen-antibody complex formation after the concentration increases. The limit of detection (LOD) was from 0.70 pmol L^−1^, which corresponds to the first detectable concentration of spike protein, as presented in the literature [45,82]. It is possible to observe the decay of the peak current and the increase in the R_ct_ with increasing protein concentration, leading to suppression of the redox probe signal. In addition, the immunosensor can be easily used as a qualitative sensor, i.e., as an on-off sensor, which presented a dissociation constant (K_d_) of 1.75 ± 0.21 pmol L^−1^ [83,84]. This is possible because the sensor application depends on its selectivity during the immunocomplex formation, independent of the protein concentration.

For EIS measurements, spike protein detection was performed at a concentration range from 1.0 to 30.0 pmol L^−1^ (Figure 5C,D). The Nyquist diagram was fitted for each measure using the Randles circuit (insert), which allows the charge transfer resistance values to be obtained. It was observed that the increase in spike protein concentration promoted the increase of the charge transfer resistance. The formation of the immune complex partially blocks the electrode surface, avoiding that part of the electrochemical probe reacting with the electrode surface. In other words, the increase of Rct values is linear to the spike protein concentration, which allows the analytical correlation between NIC% and C_Spike protein_. The LOD was from 1.0 pmol L^−1^, as the first detectable concentration.

The analytical performance of the immunosensor was compared with other immunosensors for the detection of SARS-CoV-2 presented in the literature (Table 1). In this case, the platinum immunosensor showed a comparable or superior electrochemical performance to other immunosensors for the detection of SARS-CoV-2. For example, Mehmandoust et al. [45] proposed an immunosensor for the determination of SARS-CoV-2 using metal-organic structures (UiO-66) with the incorporation of SiO_2_ nanoparticles. Although the proposed immunosensor displayed high sensitivity, the synthesis of SiO_2_@UiO-66 led to an additional modification step in the preparation of the sensor, which is related to an additional cost for the final product. In contrast, our proposed immunosensor presents a smaller number of modification steps, which can lead to a gain in preparation time and, consequently, a better final cost for the obtained device. Liv et al. [75] detected SARS-CoV-2 using a glassy carbon electrode (GCE) modified with Au-clusters, obtaining an ultrasensitive response. However, the use of GCE could restrict the application of the device as a portable sensor for point-of-care analysis. In another work, Vásquez et al. [83] detected SARS-CoV-2 using carboxylated magnetic beads functionalized with SARS-CoV-2 antibodies linked to angiotensin-converting enzyme host receptor (ACE2), additionally marked with streptavidin (poly) horseradish peroxidase (HRP) reporter enzyme. Despite the use of HRP as a signal amplifier, a second marker can lead to increased costs for preparing the immunosensor, which may affect the accessibility of the test for underdeveloped regions. In this sense, our immunosensor uses platinum itself as a signal amplifier, being confectioned in Bio-PET. In addition, allied to its low cost, this substrate provided excellent mechanical and thermal properties for the confection of flexible sensors and biosensors [20].

### 3.5. Determination of SARS-CoV-2 Spike Protein in Saliva Sample

**Figure 6:**
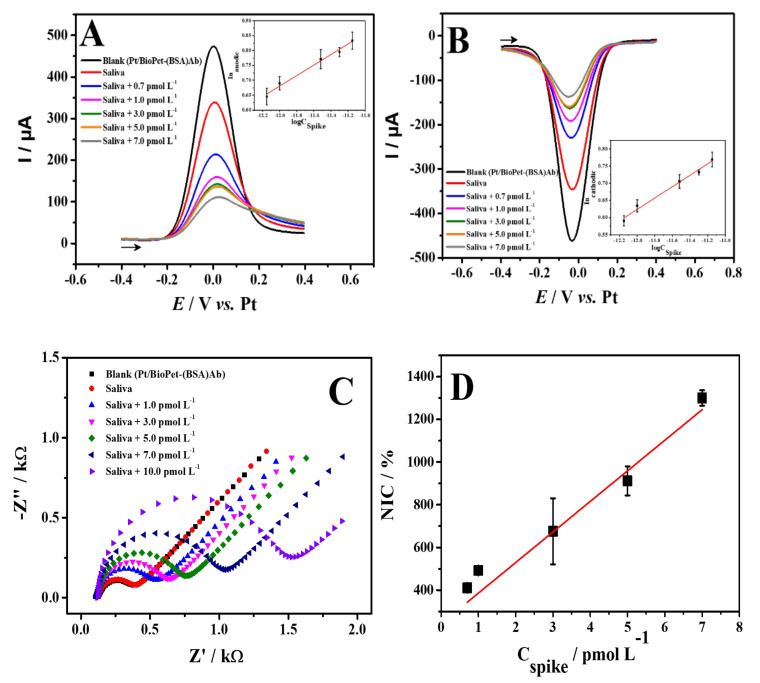
Square wave voltammograms direct (**A**) and reverse (**B**) obtained for different concentrations of spike protein of 0.7−7.0 pmol L^−1^ in the saliva sample. Inset: Plot of peak current versus spike protein concentration. SWV experimental conditions: pulse amplitude: 80 mV; frequency: 80 Hz; step potential: 10 mV. (**C**) Nyquist plots obtained for different concentrations of Spike protein of 1.0−30.0 pmol L^−1^ in the saliva sample. (**D**) Plot of spike protein concentrations versus NIC%. Supporting electrolyte: 5.0 mmol L^−1^ [Fe(CN)_6_]^3−/4−^ in 0.10 mol L^−1^ KCl (pH7.0).

Saliva as a common means of transmission of SARS-CoV-2 and other infectious diseases [90] and as a non-invasive method of analysis was used for our work. Thus, the analytical performance of the immunosensor was evaluated in synthetic saliva at known concentrations of spike protein. SWV and EIS measurements were recorded in the presence of 5.0 mmol L^−1^ [Fe(CN)_6_]^3−/4−^ at concentrations of 0.7 to 7.0 pmol L^−1^ and 1.0 to 10 pmol L^−1^ of the spike protein, respectively (Figure 6). The analytical curves obtained in saliva showed similar behavior to those in PBS media, with a linear decrease in the anodic (R^2^ = 0.983) and cathodic currents (R^2^ = 0.983) for SWV (Figure 6A,B), and a linear increase in the impedimetric response (R^2^ = 0.967, Figure 6C,D).

Thus, the applicability of the immunosensor in detecting the spike protein in artificial saliva medium has been proven, thus demonstrating its efficiency with the gold standard RT-PCR technique. In this regard, as a comparison, the study by Basso et al. [91] detected SARS-CoV-2 antigen in saliva samples using RT-PCR with an LOD of 0.2 ng mL^−1^. This shows that our immunosensor has a high sensitivity to detect the presence of the SARS-CoV-2 virus at the initial stage where the viral load is extremely low, and repeat testing is not necessary for an accurate diagnosis [92].

## 4. Conclusions

This work reports the development of an environmentally friendly sensor and immunosensor based on platinum on a flexible substrate (Pt/Bio-PET). It is important to mention that no physical or chemical pre-treatment was required for signal amplification. Consequently, the sensor presented a lower cost and time of production. Furthermore, the 3D-printed platform for the electrode connection and electrochemical analysis allowed the use of low sample volume (10 μL). This allows the application of the proposed device for point-of-care analysis. The device also showed a satisfactory detection of L-cysteine and the SARS-CoV-2 spike protein, presenting low limits of detection for both voltammetric and impedimetric techniques. Furthermore, the presented Pt/Bio-PET allowed multiplex detection of separate biomarkers, paving the way for a new generation of cost-effective, fast, and sensitive devices for clinical diagnosis and assessment of disease progression. These features make the device an alternative for the diagnosis of SARS-CoV-2-related biomarkers, allowing a rapid and precise response.

## Figures and Tables

**Table 1:** Analytical performance of the flexible label-free platinum immunosensor for SARS-CoV-2 determination compared with the literature.

Electrode	Technique	LDR	LOD	Sample	Refs.
SPCE	Chronoamperometry	0.5 to 10 ng mL^−1^	0.19 ng mL^−1^	Artificial saliva	[85]
Carbon black-SPE	SWV	0.04 to 10 μg mL^−1^	19.0 ng mL^−1^	Saliva	[86]
SPCE	EIS	1.0 × 10^−11^ to 1.0 × 10^−7^ mol L^−1^	19.0 ng mL^−1^	Human saliva	[83]
Gpt-PLA	CV	5.0 to 75 nmol L^−1^	1.36 nmol L^−1^	Artificial saliva	[87]
SiO_2_@UiO-66/SPCE	EIS	100 fg mL^−1^ to 10 ng mL^−1^	100 fg mL^−1^	Nasal fluid	[45]
GCE	SWV	0.1 a 1000 ag mL^−1^	0.01 ag mL^−1^	Saliva and oropharyngeal swab	[75]
G/PLA	EIS	1.0 to 10 μg mL^−1^	0.5 μg mL^−1^	Human serum	[88]
SPAuE	Chronoamperometry	0 e 1.0 μg mL^−1^	22.5 ng mL^−1^	Nasopharyngeal swab	[89]
Pt/BioPET	SWV; EIS	0.7 to 7.0 pmol L^−1^ 1.0 to 30.0 pmol L^−1^	0.7 pmol L^−1^ 1.0 pmol L^−1^	Artificial saliva	This Work

LDR: Linear dynamic range; LOD: Limit of detection; SPCE: screen-printed carbon electrodes; Gpt-PLA: graphite-polylactic acid; @UiO-66: universitetet i Oslo-66; GCE: glassy carbon electrode; G/PLA: graphene/polylactic acid; SPAuE: gold screen-printed electrode.

## Data Availability

The data is available upon request.

## Supplementary Materials

The following supporting information can be downloaded at: https://www.mdpi.com/article/10.3390/bios13020190/s1. Figure S1: Assembly of the proposed device in 3D printing for clinical analysis; Figure S2: (A) Flexible platinum electrodes fabricated by photolithography on Bio-PET substrate (geometric area of 0.03 ± 0.1 cm^2^); (B) Mechanical stress process applied to an electrode; Figure S3: EDS spectra for the semi-quantitative distribution of chemical elements on the platinum electrode surface; Figure S4: SEM images of Pt film thickness; Figure S5: (A) Cyclic voltammograms of the flexible platinum electrode in the presence (▬) and absence (▬) of an equimolar mixture of 5.0 mmol L^−1^ [Fe(CN)6]^3−/4−^; (B) Cyclic voltammograms obtained varying the scan rate at 10, 20, 30, 50, 70, 90, 110, 130, 160, 200 and 250 mV s^−1^; Inset: plot of current response in function of v^1/2^; Figure S6: Proposed mechanism of the oxidation of L-Cys on the surface of the Pt electrode; Figure S7: Effect of pH for the cyclic voltammetric behavior of the Pl electrode in presence of 500 μmol L^−1^ L-Cys; Figure S8: (A) Cyclic voltammograms obtained with the Pt electrode in 0.1 mol L^−1^ PBS (pH 6.0) containing 1000 μmol L^−1^ L-cysteine at different scan rates, from 0.01 to 0.30 V s^−1^. (B) Linear plot of L-cysteine Ipa versus v^1/2^ (V s^−1^)^1/2^; Figure S9: (A) Chronoamperogram and (B) current response obtained for successive additions of L-Cys and different interfering species; Figure S10: Fit simulation of the equivalent circuit for the first and last immunosensor modification step; Figure S11: Square wave voltammetry direct (A–C) and reverse (A′–C′) effect of experimental conditions as function of peak width at half height (A″–C″); Table S1: Analytical performance of the Pt-based sensor for the L-Cys determination compared with other sensors in the literature.
