# Supplementary material for: Flexible Label-Free Platinum and Bio-PET-Based Immunosensor for the Detection of SARS-CoV-2
Source: Biosensors (Basel). 2023 Jan 26;13(2):190. doi: 10.3390/bios13020190 (PMC9954080; doi:10.3390/bios13020190)
Supplement: Supplementary file 1 [file biosensors-13-00190-s001.zip › biosensors-2097530-supplementary.pdf]

Supporting information:

Flexible label-free platinum and Bio-PET-based immunosensor for the detection of SARS-CoV-2

Rodrigo Vieira Blasques[1,2], Paulo Roberto de Oliveira[1,3], Cristiane Kalinke[3,4], Laís Canniatti Brazaca[5], Robert D. Crapnell[3], Juliano Alves Bonacin[4], Craig E. Banks[3], Bruno Campos Janegitz[1]*

[1]Laboratory of Sensors, Nanomedicine and Nanostructured Materials, Federal University of São Carlos, Araras,13600-970, Brazil.

[2] Department of Physics, Chemistry, and Mathematics, Federal University of São Carlos, Sorocaba, São Paulo, Brazil, 18052-780

[3] Faculty of Science and Engineering, Manchester Metropolitan University, Chester Street, Manchester, M1 5GD, United Kingdom.

[4] Institute of Chemistry, University of Campinas, 13083-970, Campinas, SP, Brazil

[5] Department of Chemistry and Chemical Biology, Harvard University, Cambridge, Massachusetts, 02138, United States.

* Corresponding authors: brunocj@ufscar.br (Bruno Campos Janegitz);

paulo.oliveira@ufscar.br (Paulo Roberto de Oliveira).

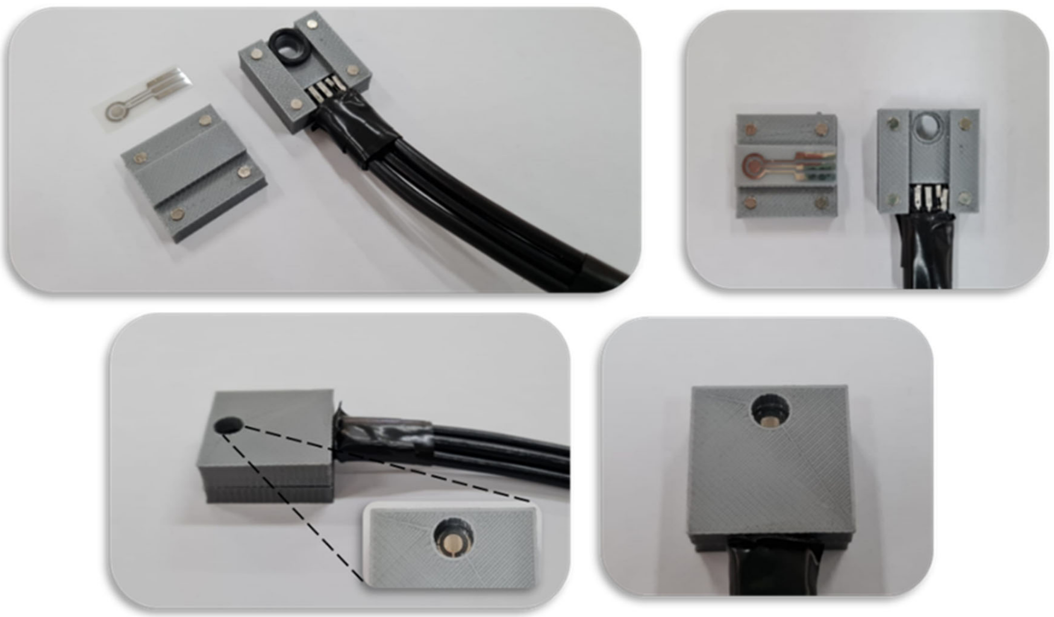

Figure S1. Assembly of the proposed device in 3D printing for clinical analysis.

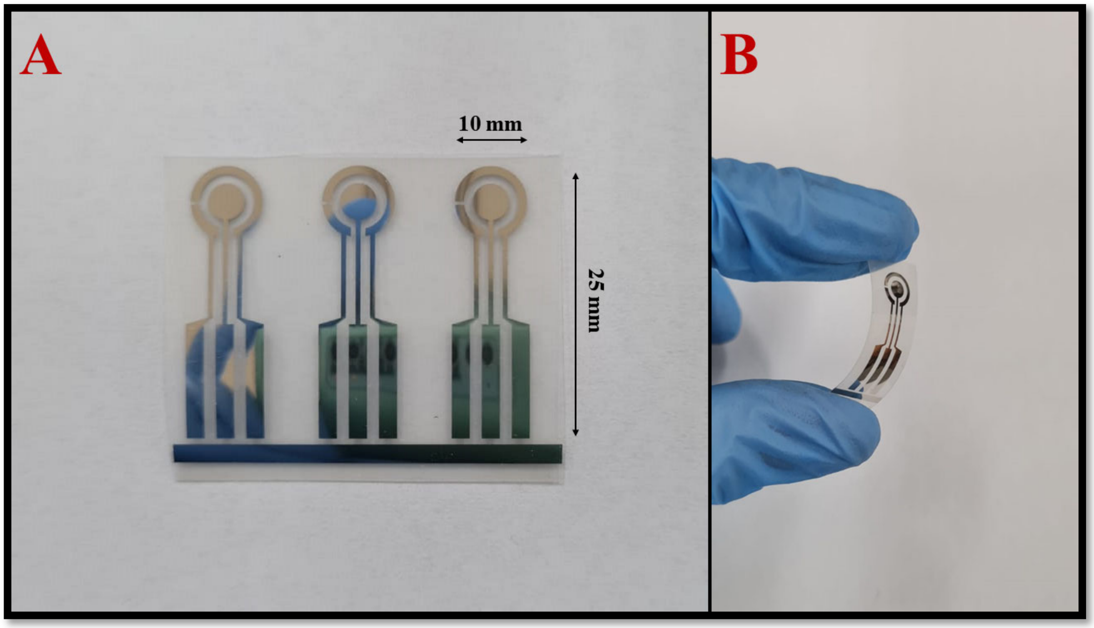

Figure S2. (A) Flexible platinum electrodes fabricated by photolithography on Bio-PET substrate (geometric area of 0.03 ± 0.1 cm^2); (B) Mechanical stress process applied to an electrode.

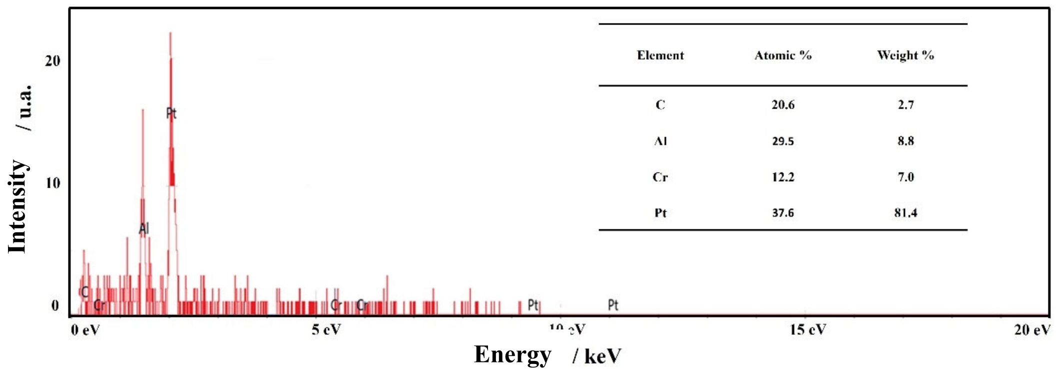

Figure S3. EDS spectra for the semi-quantitative distribution of chemical elements on the platinum electrode surface.

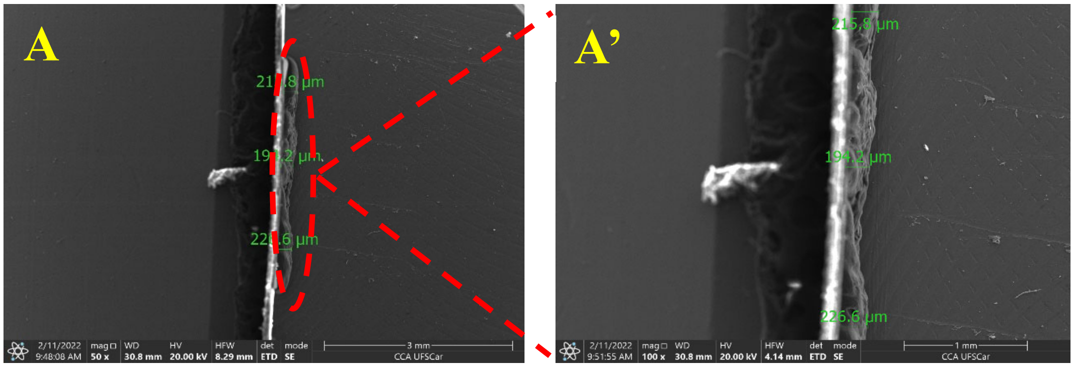

Figure S4. SEM images of Pt film thickness.

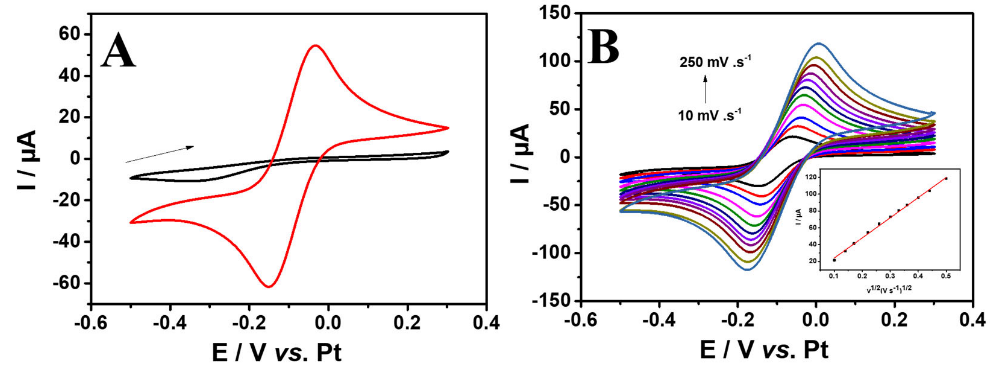

Figure S5. (A) Cyclic voltammograms of the flexible platinum electrode in the presence (▬) and absence (▬) of an equimolar mixture of 5.0 mmol L^{-1} $[Fe(CN)_6]^{3-/4-}$; (B) Cyclic voltammograms obtained varying the scan rate at 10, 20, 30, 50, 70, 90, 110, 130, 160, 200 and 250 mV s^{-1}; Inset: plot of current response in function of $v^{1/2}$.

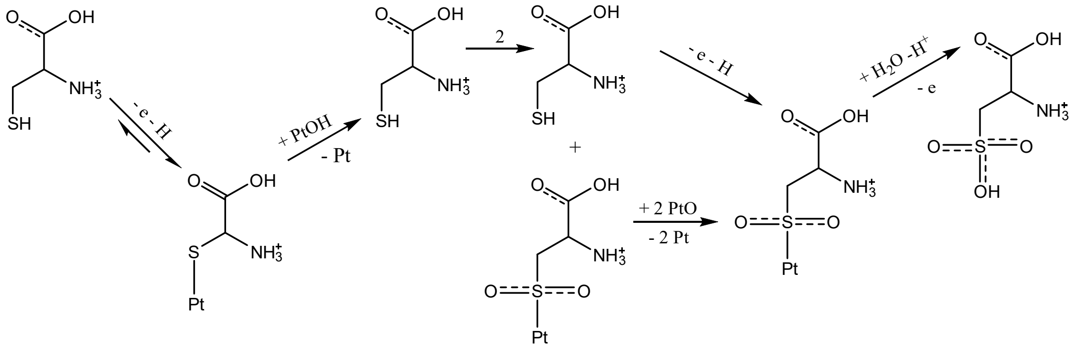

Figure S6. Proposed mechanism of the oxidation of L-Cys on the surface of the Pt electrode [1].

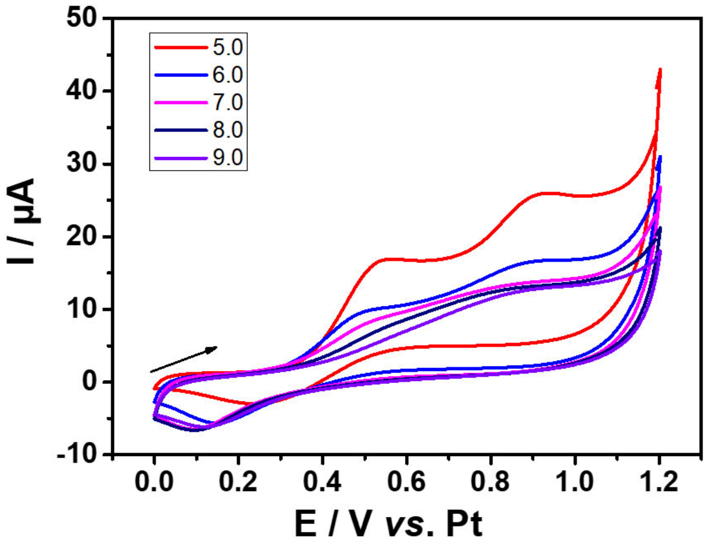

Figure S7. Effect of pH for the cyclic voltammetric behavior of the Pl electrode in presence of 500 µmol L^{-1} L-Cys.

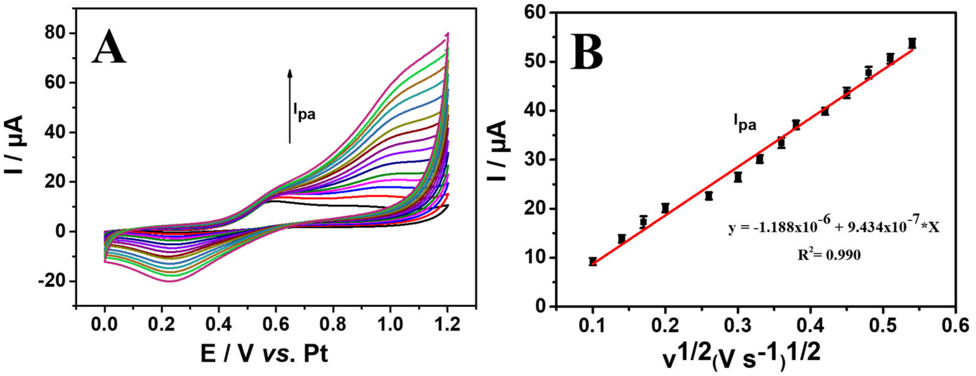

Figure S8. (A) Cyclic voltammograms obtained with the Pt electrode in 0.1 mol L^{-1} PBS (pH 6.0) containing 1000 µmol L^{-1} L-cysteine at different scan rates, from 0.01 to 0.30 V s^{-1}. (B) Linear plot of L-cysteine I_{pa} *versus* $v^{1/2}$ $(V\ s^{-1})^{1/2}$.

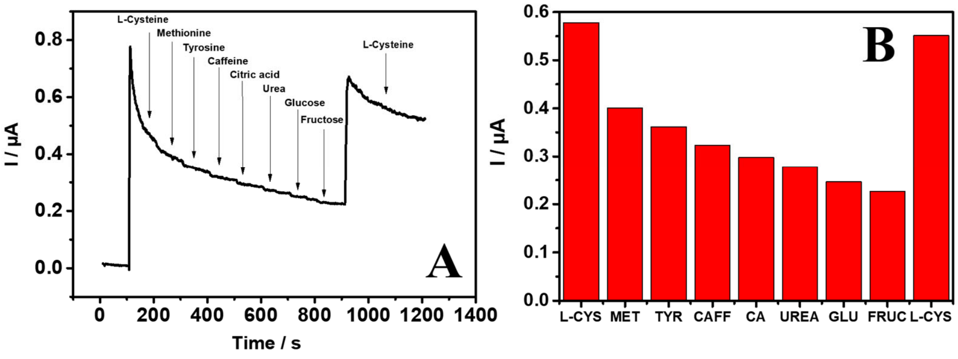

Figure S9. (A) Chronoamperogram and (B) current response obtained for successive additions of L-Cys and different interfering species (Methionine, Tyrosine, Caffeine, Citric acid, Urea, Glucose, and Fructose) at an applied potential of 0.55 V. Supporting electrolyte: 0.1 mol L^{-1} PBS pH 6.0.

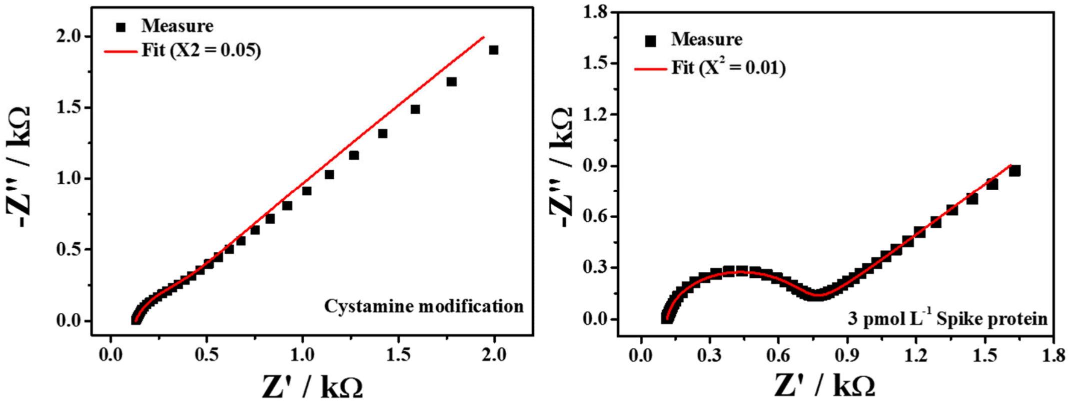

Figure S10. Fit simulation of the equivalent circuit for the first and last immunosensor modification step.

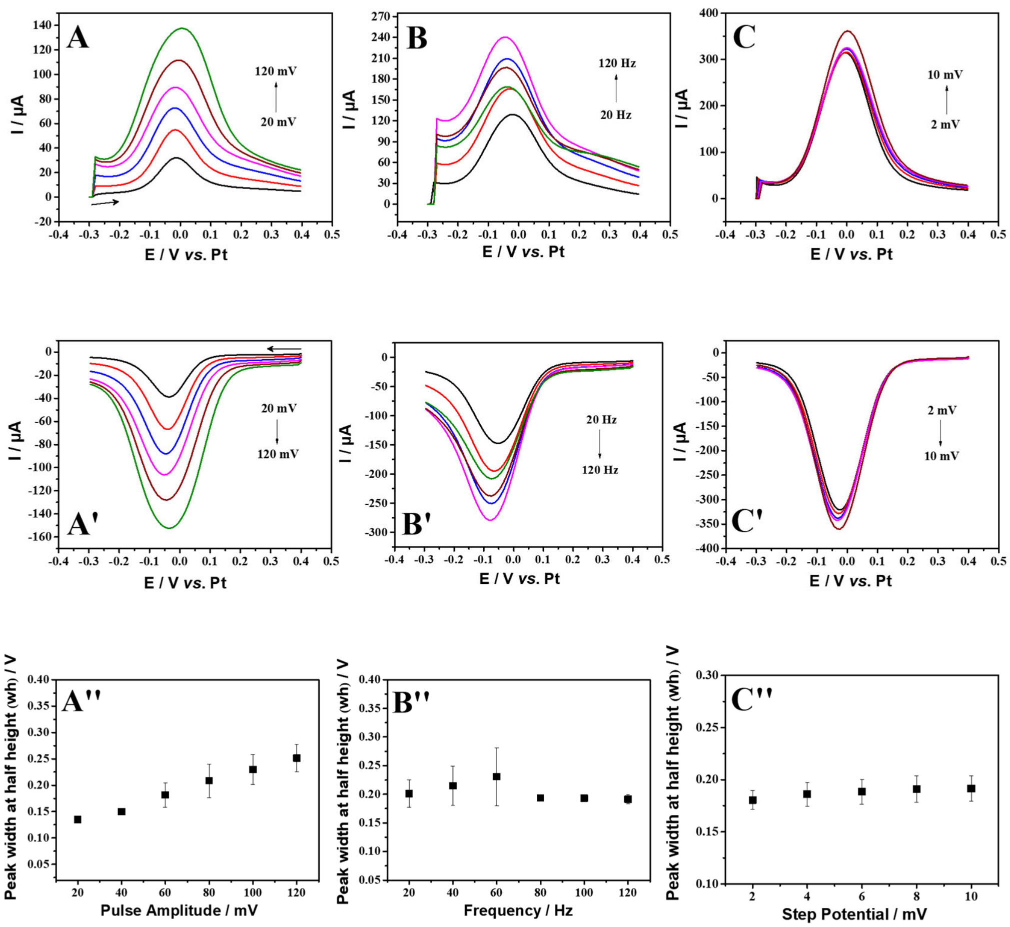

Figure S11. Square wave voltammetry direct (A-C) and reverse (A’-C’) effect of experimental conditions as function of peak width at half height (A’’-C’’) for 0.1 µmol L^{-1} Spike protein in PBS 1x in the presence equimolar mixture of 5.0 mmol L^{-1} $[Fe(CN)_6]^{3-/4-}$; pulse amplitude from 20 to 120 mV (A’’), frequency from 20 to 120 Hz (B’’) and step potential from 2 to 10 mV (C’’).

Table S1 - Analytical performance of the Pt-based sensor for the L-Cys determination compared with other sensors in the literature.

Electrode	Technique	Linear range (µmol L^{-1})	LOD (µmol L^{-1})	Ref.
AuNP/MnP/FTO	Chronoamperometry	12.0 to 34.0	2.40	[2]
SPE/PB-ammine	Chronoamperometry	100 to 500	72.0	[3]
GR/CD/Pt/SPE	DPV	0.50 to 40.0<br>40.0 to 170	0.12	[4]
Poli (ácido *p* -cumárico) / MWNT/GCE	DPV	7.5 to 1000	1.1	[5]
$CuFe_2O_4$/rGO-Au	CV	50.0 to 400	0.383	[6]
3D PLA-G_{DMF-EC}+PB	Chronoamperometry	3.0 to 230	0.858	[7]
FePc–AuNP/GPE	DPV	50.0 to 1000	0.27	[8]
GC/RGO/cobalt (II) porphyrazine	Chronoamperometry	1000 to 6.6×10^5	0.79	[9]
MoN/N-MWNTs	Chronoamperometry	5.0 to 1.26×10^4	3.64	[10]
Cu-CoHCF	CV	6.0 to 1000	5.0	[11]
InHCF	Chronoamperometry	100 to 1000	50	[12]
CoTAPc-MWNTs	Chronoamperometry	5.0 to 40	0.28	[13]
Pt/Bio-PET	*Chronoamperometry*	*3.98 to 39.0*<br>*39.0 to 145*	*0.70*<br>*2.36*	*This work*

Notes: AuNP: gold nanoparticles; MnP: metallated porphyrin; FTO: fluorine tin oxide-coated glass; SPE: screen-printed electrodes; PLA-G_{DMF-EC}: activated polylactic acid and graphene electrode; PB: Prussian blue; GR: reduced graphene; CD: oxide-β-cyclodextrin; Pt: platinum; MWNT: multi-walled carbon nanotubes; GCE: glassy carbon electrode; $CuFe_2O_4$: copper ferrite;

rGO: reduced graphene oxide; MoN/N-MWNTs: Molybdenum nitride/nitrogen-doped multi-walled carbon nanotubes; Cu-CoHCF: copper–cobalt hexacyanoferrate; InHCF: Indium hexacyanoferrate; CoTAPc: cobalt tetraaminophthalocyanine

References

[1] A.H.B. Dourado, M. Arenz, S.I. Córdoba de Torresi, Mechanism of Electrochemical L-Cysteine Oxidation on Pt, 6(2019) 1009-13.

[2] M.C. Gallo, B.M. Pires, K.C.F. Toledo, S.A.V. Jannuzzi, E.G.R. Arruda, A.L.B. Formiga, et al., The use of modified electrodes by hybrid systems gold nanoparticles/Mn-porphyrin in electrochemical detection of cysteine, Synthetic Metals, 198(2014) 335-9.

[3] J.A. Bonacin, P.L. Dos Santos, V. Katic, C.W. Foster, C.E. Banks, Use of Screen-printed Electrodes Modified by Prussian Blue and Analogues in Sensing of Cysteine, Electroanalysis, 30(2018) 170-9.

[4] M. Singh, N. Jaiswal, I. Tiwari, C.W. Foster, C.E. Banks, A reduced graphene oxide-cyclodextrin-platinum nanocomposite modified screen printed electrode for the detection of cysteine, Journal of Electroanalytical Chemistry, 829(2018) 230-40.

[5] G. Ziyatdinova, E. Kozlova, H. Budnikov, Selective electrochemical sensor based on the electropolymerized p-coumaric acid for the direct determination of l-cysteine, Electrochimica Acta, 270(2018) 369-77.

[6] K. Atacan, CuFe2O4/reduced graphene oxide nanocomposite decorated with gold nanoparticles as a new electrochemical sensor material for L-cysteine detection, Journal of Alloys and Compounds, 791(2019) 391-401.

[7] C. Kalinke, P.R. de Oliveira, B.C. Janegitz, J.A. Bonacin, Prussian blue nanoparticles anchored on activated 3D printed sensor for the detection of L-cysteine, Sensors and Actuators B: Chemical, 362(2022) 131797.

[8] M.N. Abbas, A.A. Saeed, B. Singh, A.A. Radowan, E. Dempsey, A cysteine sensor based on a gold nanoparticle–iron phthalocyanine modified graphite paste electrode, Analytical Methods, 7(2015) 2529-36.

[9] M. Falkowski, T. Rebis, M. Kryjewski, L. Popenda, S. Lijewski, S. Jurga, et al., An enhanced electrochemical nanohybrid sensing platform consisting of reduced graphene oxide and sulfanyl metalloporphyrazines for sensitive determination of hydrogen peroxide and l-cysteine, Dyes and Pigments, 138(2017) 190-203.

[10] D. Geng, M. Li, X. Bo, L. Guo, Molybdenum nitride/nitrogen-doped multi-walled carbon nanotubes hybrid nanocomposites as novel electrochemical sensor for detection l-cysteine, Sensors and Actuators B: Chemical, 237(2016) 581-90.
[11] A. Abbaspour, A. Ghaffarinejad, Electrocatalytic oxidation of l-cysteine with a stable copper–cobalt hexacyanoferrate electrochemically modified carbon paste electrode, Electrochimica Acta, 53(2008) 6643-50.
[12] K.-S. Tseng, L.-C. Chen, K.-C. Ho, Amperometric Detection of Cysteine at an In3+ Stabilized Indium Hexacyanoferrate Modified Electrode, 18(2006) 1306-12.
[13] S. Nyoni, T. Mugadza, T. Nyokong, Improved l-cysteine electrocatalysis through a sequential drop dry technique using multi-walled carbon nanotubes and cobalt tetraaminophthalocyanine conjugates, Electrochimica Acta, 128(2014) 32-40.
